# Virulence factors, antifungal susceptibility and molecular mechanisms of azole resistance among *Candida parapsilosis* complex isolates recovered from clinical specimens

**DOI:** 10.1186/s12929-017-0376-2

**Published:** 2017-09-04

**Authors:** Sourour Neji, Ines Hadrich, Houaida Trabelsi, Salma Abbes, Fatma Cheikhrouhou, Hayet Sellami, Fattouma Makni, Ali Ayadi

**Affiliations:** 1 Laboratory of Parasitology – Mycology, UH Habib Bourguiba, Sfax, Tunisia; 2 Laboratory of Fungal and Parasitic Molecular Biology, School of Medicine, University of Sfax, Sfax, Tunisia

**Keywords:** *Candida parapsilosis* Complex, Virulence factors, Proteases, Phospholipases, Haemolysin, Biofilm production, Antifungal susceptibility, Mechanisms of resistance

## Abstract

**Background:**

The aim of this study was to determine the biofilm formation, the extracellular enzymatic activities of 182 clinical isolates of the *Candida parapsilosis* complex.

**Methods:**

Molecular identification of the *C. parapsilosis* species complex was performed using PCR RFLP of SADH gene and PCR sequencing of ITS region. The susceptibility of ours isolates to antifungal agents and molecular mechanisms underlying azole resistance were evaluated.

**Results:**

63.5% of *C. parapsilosis* were phospholipase positive with moderate activity for the majority of strains. None of the *C. metapsilosis* or *C. orthopsilosis* isolates was able to produce phospholipase. Higher caseinase activities were detected in *C. parapsilosis* (Pz = 0.5 ± 0.18) and *C. orthopsilosis* (Pz = 0.49 ± 0.07) than in *C. metapsilosis* isolates (Pz = 0.72 ± 0.1). 96.5% of *C. parapsilosis* strains and all isolates of *C. metapsilosis* and *C. orthopsilosis* produced gelatinase. All the strains possessed the ability to show haemolysis on blood agar. *C. metapsilosis* exhibited the low haemolysin production with statistical significant differences compared to *C. parapsilosis* and *C. orthopsilosis*. The biofilm forming ability of *C. parapsilosis* was highly strain dependent with important heterogeneity, which was less evident with both *C. orthopsilosis* and *C. metapsilosis*.

Some *C. parapsilosis* isolates met the criterion for susceptible dose dependent to fluconazole (10.91%), itraconazole (16.36%) and voriconazole (7.27%). Moreover, 5.45% and 1.82% of *C. parapsilosis* isolates were respectively resistant to fluconazole and voriconazole. All strains of *C. metapsilosis* and *C. orthopsilosis* were susceptible to azoles; and isolates of all three species exhibited 100% of susceptibility to caspofungin, amphotericin B and 5-flucytosine.

**Conclusions:**

A combination of molecular mechanisms, including the overexpression of ERG11, and genes encoding efflux pumps (CDR1, MDR1, and MRR1) were involved in azole resistance in *C. parapsilosis*.

## Background

The *Candida parapsilosis* complex has emerged as an opportunistic fungal pathogen especially notable for causing nosocomial infections worldwide. It is composed of three genetically distinct species, namely *C. parapsilosis* sensu stricto, *C. orthopsilosis and C. metapsilosis*, which are physiologically and morphologically indistinguishable [54]. Previous data have shown that these three species exhibit different prevalence rates, virulence, and in vitro antifungal susceptibility [3, 23, 56, 59].

Many virulence factors contribute to the pathogenesis of candidiasis, allowing the fungal cells to escape and/or overcome the host defenses. Among these factors proposed in the literature, adherence to host cells and/or tissues as well as to inert supports, phenotypic switching, biofilm formation and secretion of a large array of hydrolytic enzymes are included [1, 45, 47, 60]. But differences in these virulence factors among *C. parapsilosis* complex species have not been widely investigated. Aggravating this scenario, many contra-dictory results have been generated [64]. So, further studies are needed to better understand the characteristics, including putative virulence traits, drug resistance trends, especially of the two rarely isolated species, *C. orthopsilosis* and *C. metapsilosis*. A better knowledge could have clinical relevance, as it may be useful in guiding therapeutic decisions [13].

The aim of our study was to investigate the distribution of five virulence factors namely: biofilm production, caseinase, gelatinase, phospholipase and haemolysin extracellular production among *C. parapsilosis* complex isolates. The association of these virulence factors with resistance to antifungal agents was studied and the susceptibility of ours isolates to antifungal agents was evaluated in vitro*.* Additional aim of this study was to assess the relative contribution of the number of copies of drug resistance genes and their overexpressions to the azole resistance of *C. parapsilosis*.

## Methods

### Fungal strains

A total number of 182 *C. parapsilosis* complex species isolates included in this study were recovered from clinical samples received by Department of Parasitology – Mycology-university hospital - Sfax, Tunisia, during a 14 year period (from January 2002 to January 2016). They were 172 strains of *C. parapsilosis* sensu stricto*,* 6 strains of *C. metapsilosis* and 4 strains of *C. orthopsilosis.* Isolates were identified to the species level by standard methods. Molecular identification of the *C. parapsilosis* species complex was performed by *Ban*I PCR RFLP of SADH gene according to Tavanti A et al. [54], and supplemented, as needed by internal transcribed spacer 1 (ITS1), 5.8S, and ITS2 region rRNA sequence analysis [62]. Ten of these isolates were as reference strains, identified by ITS sequence analysis of the ribosomal DNA, and deposed in GenBank: *C. parapsilosis* sensu stricto (KT948326), *C. metapsilosis* (KU665248, KU665249, KU665250, KU665251 and KU665252) and *C. orthopsilosis* (KU665253, KU665254, KU665255, and KU665256).

Seven reference strains were included: *C. parapsilosis* (ATCC 22019), *C. orthopsilosis* (1,219,482, 1,343,124), *C. metapsilosis* (1240011), *C. albicans* (ATCC 3153), *C. glabrata* (CBS 138) and *C. tropicalis* (ATCC 66029).

### Phospholipase activity

Phospholipase activity of *Candida* species was detected by egg yolk agar method [40]. The egg yolk medium consisted of 65 g Sabouraud dextrose agar (SDA), 55.3 g NaCl, 5.5 g CaCl2 and 10% sterile egg yolk. Ten microliters of saline suspension prepared from a 48 h yeast culture (approximately 10^6^ cells/ml determined through densitometer) was spot inoculated in triplicate onto the medium and incubated at 37 °C for 7 days. The diameter of the colony (*a*) and the diameter of the precipitation zone plus the diameter of the colony (*b*) were measured. Phospholipase index was designated as Pz = *a*/*b*, as described by Price et al. [40]. According to this definition, low Pz values mean high enzymatic production and, inversely, high Pz values indicate low enzymatic production. The enzymatic activity was scored into four categories: a Pz of 1.0 indicated no enzymatic activity; a Pz between 0.99 and 0.90 indicated weak enzymatic activity; Pz between 0.89 and 0.70 corresponded to moderate activity; and low Pz values ≤0.69 meant strong enzymatic activities.

### Proteinase activity

For testing the proteinase activity of the *Candida* isolates, caseinase, and gelatinase activity tests were performed. A standard inoculum (10^6^ cells/ml) was prepared in saline solution from a 48 h yeast culture for each isolate.

### Caseinase activity

Caseinase activity was measured by single diffusion technique in SDA agar plates provided with 1% casein [41]. Three aliquots of 10 μl of standard inoculum were spotted for each strain. The plate was then incubated at 37 °C for 5 days. The zone of clearance was measured by standard procedures.

### Gelatinase activity

Gelatinase assay was done on SDA agar plates prepared with 1% gelatin [41]. Single diffusion technique was applied in triplicate. The plate was then incubated for 5 days at 37 °C. The appearance of inhibition zone was clearly visualized by the addition of 0.1% mercuric chloride. The zone diameter was measured by standard procedures [2].

### Haemolytic activity

Haemolysin assay for *Candida* strains was performed according to a previously validated protocol by Luo G et al. [31]. In brief, Sabouraud dextrose agar supplemented with 6% human blood and 3% glucose (pH = 5.6) was used to determine the hemolysin production. Suspension of yeast (10^6^ cells/ml) was prepared in saline solution and 10 μl was spot inoculated on human blood agar plates, incubated at 37 °C in 5% CO2 for 5 days. The haemolytic activity was calculated by dividing the diameters of the colony and the translucent zone of haemolysis.

### Biofilm formation

The in vitro Biofilm formation of *Candida parapsilosis* complex isolates was quantified by a modification of a crystal violet assay as described by Silva S et al. [50] with some modifications. Briefly, *Candida* isolates were first cultured at 37 °C for 24 h on SDA plates. 200 μl of standardized cell suspensions (containing 1 _ 10^6^ cells ml ^−1^ in yeast peptone galactose medium (YPG)) were trans-ferred to each well of 96-well polystyrene microtiter plates (Kartell. SPA, Italy) and incubated at 37 °C on a shaker at 120 rpm/min. As a negative control, test medium without cells was added to three wells of each plate. At 24 h, 50 μl of YPG medium was added. The preparations were then incubated for a further 48 h. After the adhesion stage, non-adherent cells were removed by washing the wells twice with sterile ultra-pure water. Biofilms were fixed with 250 μl of methanol, which was removed after 15 min of contact. The microtiter plates were dried at room temperature, and 250 μl of crystal violet (CV) (1% *v*/v) added to each well and incubated for 5 min. The wells were then gently washed with sterile, ultra-pure water and 250 μl of acetic acid added to release and dissolve the stain. The absorbance of the obtained solution was read in triplicate in a microtiter plate reader (Halo LED 96, Dynamica, EU) at 620 nm. The absorbance values for the controls (containing no cells) were subtracted from the values for the test wells to eliminate spurious results due to background interference. Data were recorded as arithmetic means of absorbance values. Experiments were repeated as part of three independent assays.

### Antifungal susceptibility testing

Antifungal susceptibility testing was performed as part of routine patient care. The in vitro susceptibility to eight antifungal drugs (fluconazole, itraconazole, ketoconazole, posaconazole, voriconazole, caspofungin, amphotericin B and 5-flucytosine) was determined using Sensititre Yeast OneTM YO8 methodology (Trek Diagnostic Systems) following the manufacturer’s Instructions. minimal inhibitory concentration (MIC) values were interpreted according to the M27-A3 and M27-S4 documents published by the Clinical and Laboratory Standards Institute (CLSI) (CLSI 2008, CLSI 2012) [38, 39]. The clinical breakpoints defined for *Candida parapsilosis* were used for the interpretation of minimum inhibitory concentration (MIC) data as follows: susceptible (S) ≤2 μg/ml, susceptible dose dependent (SDD) 4 μg/ml, resistant (R) ≥8 μg/ml for fluconazole; S ≤ 0.125 μg/ml, SDD 0.25–0.5 μg/ml, *R* ≥ 1 μg/ml for itraconazole; S ≤ 0.125 μg/ml, S-DD 0.25–0.5 μg/ml, *R* ≥ 1 μg/ml for voriconazole; S ≤ 2 μg/ml, intermediate (I) 4 μg/ml, *R* ≥ 8 μg/ml for caspofungin; and S ≤ 4 μg/ml, I 8–16 μg/ml, *R* ≥ 32 μg/ml for 5-flucytosine. For amphotericin B, we adopted putative breakpoints as S, ≤1 mg/l and R, >1 μg/ml [37].

### Mechanisms of azole resistance

A RT-qPCR assay was developed to explore mechanisms of *C. parapsilosis* azole resistance. The levels of mRNA and DNA of the tested genes (ERG11, CDR1, MDR1, and MRR1) and the actin reference gene (ACT1) were measured. The primers and probes were designed using Primer3 software (http://bioinfo.ut.ee/primer3-0.4.0/) and are summarized in Table 1.

**Table 1:** The sequences of primers and probes used in RT-qPCR

Gene		Primers and probes
ACT1	F	5′- CGAACGTGGTTACGGTTTCT- 3’
	R	5′- TGACCATCTGGCAATTCGTA - 3’
	Probe	TET-TGCAAACCTCATCACAATCA-MGB
CDR1	F	5′- GCTGTTGATCAAAGGGGTGT - 3’
	R	5′- ATCCAAAATCCAGGCAACTG - 3’
	Probe	FAM- CTGATAATGCCGCCAATCTT-MGB
ERG11	F	5′- TGTTGCATTTGGCTGAGAAG - 3’
	R	5′- TCTGAGGGTTTCCTTGATGG - 3’
	Probe	FAM-GGTAAAGGTGGCAACTTGGA-MGB
MDR1	F	5′- TCCCCATTGCTATTGTTGGT - 3’
	R	5′- TGCGCCCATATAATTGAACA - 3’
	Probe	FAM- TTGGTCGGCAACGACATATA-MGB
MRR1	F	5′- CAGCTGCAACAACCACAACT - 3’
	R	5′- TATCATCTAGGCCGCCATTC - 3’
	Probe	FAM- GCAACCACAGCCTATAGGGA-MGB

Cellular lysates were prepared from cells grown in culture to mid-log phase using proteinase K (Qiagen®). RNA was extracted from cellular lysates using the RNeasy Mini Kit (Qiagen®, Germany) according to the manufacturer s’ instructions, and treated with DNase (Promega). For cDNA synthesis, 2.5 μl of total RNA was used as a template and subsequent reverse transcription was performed using the PrimeScript RT Reagent Kit (Perfect Real Time) from TaKaRa (Shiga, Japan), following the manufacturer’s instructions.

The reaction mixture (20 μl) for TaqMan assay contained 10 μl TaqMan Universal PCR Master Mix (Applied Biosystems, UK), 20 pmol of forward and reverse primers, 7 pmol of hydrolysis probe and 1 μl of of template (extracted DNA or cDNA). The thermal conditions were as follows: initial holding stage at 50 °C for 2 min and 95 °C for 10 min, followed by 50 cycles at 95 °C for 15 s and a final step at 54 °C for 1 min. All reactions were performed in triplicate in 48-well reaction plates using a StepOne™ Real Time PCR machine (Applied Biosystems).

The software StepOne™ version 2.1 (Applied Biosystems) was used to collect Cq data and to calculate the relative quantification (RQ). Fold changes in target gene expression were then normalized to reference gene via the published comparative 2^–∆∆Cq^ method according to the formula: RQ = 2^–(Cq target – Cq reference) tested – (Cq target–Cq reference) control^. A change of 2.5 times was considered as gene overexpression or an increase in gene copy number [26, 63].

### Statistical analysis

All statistical analyses were performed using IBM SPSS software (version 20.0; IBM SPSS Inc., New York, USA). Categorical variables were compared using the ×2 or Fisher’s exact test, and continuous variables by the ANOVA test. One-way ANOVA followed by Tukey’s post-hoc test were used to evaluate the level of statistical significance of clustering. A *P* value of 0.05 was considered significant. Pearson’s correlation coefficient (*r*) was calculated to measure correlation between different virulence factors. Where the value *r* = 1, means a perfect positive correlation, the value *r* = 0, means no correlation and the value *r* = −1, means a perfect negative correlation.

## Results

In our study, we evaluated the in vitro capacities of 172 *C. parapsilosis* isolates, 6 C. *metapsilosis* isolates, 4 C. *orthopsilosis* isolates and 32 *C. albicans* isolates to produce phospholipase, hemolysin and proteases (caseinase and gelatinase enzyme). Enzymatic activities of tested isolates were expressed as mean ± SD (Table 2), and activity distributions were summarized in Table 2. All the fungal strains were able to produce at least two types of hydrolytic enzymes. Table 3 presented the data from the variance analyses of the different clinical samples from which the strains were isolated and the levels of the different virulence factors.

**Table 2:** Level of phospholipase, caseinase, gelatinase and hemolysin production by *C. parapsilosis* complex species and *C. albicans*

	Phospholipase activity (number of isolates /rate of isolates)					Caseinase activity (number of isolates /rate of isolates)					Gelatinase activity (number of isolates /rate of isolates)					Hemolysin activity (number of isolates /rate of isolates)				
	Mean (*Pz*) ± SD	Strong *n* (%)	Moderate *n* (%)	Weak *n* (%)	Nul *n* (%)	Mean (*Pz*) ± SD	Strong *n* (%)	Moderate *n* (%)	Weak *n* (%)	Nul *n* (%)	Mean (*Pz*) ± SD	Strong	Moderate	Weak	nul	Mean (*Pz*) ± SD	Strong	Moderate	Weak	nul
*C.parapsilosis* Complex (*n* = 182)	0.86 ±0.13	16 (8.8)	92 (50.5)	3 (1.6)	71 (39)	0.51 ±0.18	156 (85.7)	10 (5.5)	1 (0.5)	15 (8.2)	0.71 ±0.09	85 (46.7)	91 (50)	0 (0)	6 (3.3)	0.62 ±0.1	141 (77.5)	41 (22.5)	0 (0)	0 (0)
*C.parapsilosis* (*n* = 172)	0.85 ±0.12	16 (9.3)	92 (53.5)	3 (1.7)	61 (35.5)	0.5 ±0.18	150 (87.2)	6 (3.5)	1 (0.6)	15 (8.7)	0.71 ±0.09	81 (47.1)	85 (49.4)	0 (0)	6 (3.5)	0.61 ±0.09	137 (79.7)	35 (20.3)	0 (0)	0 (0)
*C.metapsilosis* (*n* = 6)	1 ±0	0 (0)	0 (0)	0 (0)	6 (100)	0.72 ±0.1	2 (33.3)	4 (66.7)	0 (0)	0 (0)	0.72 ±0.11	3 (50)	3 (50)	0 (0)	0 (0)	0.81 ±0.08	1 (16.7)	5 (83.3)	0 (0)	0 (0)
*C.orthopsilosis* (*n* = 4)	1 ±0	0 (0)	0 (0)	0 (0)	4 (100)	0.49 ±0.07	4 (100)	0 (0)	0 (0)	0 (0)	0.71 ±0.04	1 (25)	3 (75)	0 (0)	0 (0)	0.63 ±0.09	3 (25)	1 (75)	0 (0)	0 (0)
*C.albicans* (*n* = 32)	0.62 ±0.16	26 (81.3)	2 (6.3)	0 (0)	4 (12.5)	0.69 ±0.12	19 (59.4)	9 (28.1)	4 (12.5)	0 (0)	0.72 ±0.04	7 (21.9)	25 (78.1)	0 (0)	0 (0)	0.7 ±0.11	15 (46.9)	16 (50)	1 (3.1)	0 (0)

N: number of tested isolates

n: number of isolates with positive activity for the corresponding hydrolytic enzyme

SD: standard deviation

**Table 3:** Distribution of enzymatic activities from *C. parapsilosis* complex species isolated from different clinical sites

	Phospholipase activity Mean (*Pz*) ± SD			Caseinase activity Mean (*Pz*) ± SD			Gelatinase activity Mean (*Pz*) ± SD			Hemolysin activity Mean (*Pz*) ± SD		
	*C.parapsilosis* (n/N)	*C.metapsilosis* (n/N)	*C.orthopsilosis* (n/N)	*C.parapsilosis* (n/N)	*C.metapsilosis* (n/N)	*C.orthopsilosis* (n/N)	*C.parapsilosis* (n/N)	*C.metapsilosis* (n/N)	*C.orthopsilosis* (n/N)	*C.parapsilosis* (n/N)	*C.metapsilosis* (n/N)	*C.orthopsilosis* (n/N)
Blood (*N* = 64)	0.82 ± 0.12 (46/62)	1 (0/1)	1 (0/1)	0.46 ± 0.13 (60/62)	0.71 (1/1)	0.49 (1/1)	0.7 ± 0.07 (61/62)	0.65 (1/1)	0.65 (1/1)	0.6 ± 0.09 (62/62)	0.87 (1/1)	0.73 (1/1)
Urine (*N* = 31)	0.89 ± 0.12 (16/29)	1 (0/1)	1 (0/1)	0.52 ± 0.21 (25/29)	0.67 (1/1)	0.46 (1/1)	0.71 ± 0.11 (27/29)	0.8 (1/1)	0.73 (1/1)	0.62 ± 0.1 (29/29)	0.86 (1/1)	0.65 (1/1)
Auricular sample (*N* = 43)	0.88 ± 0.12 (22/39)	1 ± 0 (0/3)	1 (0/1)	0.59 ± 0.22 (31/39)	0.73 ± 0.15 (3/3)	0.59 (1/1)	0.71 ± 0.08 (38/39)	0.74 ± 0.14 (3/3)	0.75 (1/1)	0.62 ± 0.08 (39/39)	0.75 ± 0.09 (3/3)	0.51 (1/1)
Respiratory tract (*N* = 4)	0.88 ± 0.15 (2/4)	0 (0)	0 (0)	0.45 ± 0.05 (4/4)	0 (0)	0 (0)	0.67 ± 0.04 (4/4)	0 (0)	0 (0)	0.62 ± 0.08 (4/4)	0 (0)	0 (0)
Catheter (*N* = 9)	0.88 ± 0.12 (5/9)	0 (0)	0 (0)	0.39 ± 0.03 (9/9)	0 (0)	0 (0)	0.68 ± 0.02 (9/9)	0 (0)	0 (0)	0.61 ± 0.1 (9/9)	0 (0)	0 (0)
Skin (*N* = 8)	0.82 ± 0.14 (5/7)	1 (0/1)	0 (0)	0.38 ± 0.04 (7/7)	0.72 (1/1)	0 (0)	0.71 ± 0.14 (6/7)	0.67 (1/1)	0 (0)	0.6 ± 0.11 (7/7)	0.84 (1/1)	0 (0)
Nails (*N* = 2)	1 ± 0 (0/2)	0 (0)	0 (0)	0.44 ± 0.02 (2/2)	0 (0)	0 (0)	0.78 ± 0.01 (2/2)	0 (0)	0 (0)	0.5 ± 0.01 (2/2)	0 (0)	0 (0)
Oral cavity (*N* = 3)	0.84 ± 0.07 (3/3)	0 (0)	0 (0)	0.49 ± 0.13 (3/3)	0 (0)	0 (0)	0.7 ± 0.03 (3/3)	0 (0)	0 (0)	0.57 ± 0.07 (3/3)	0 (0)	0 (0)
Vagina (*N* = 1)	0.89 (1/1)	0 (0)	0 (0)	0.35 (1/1)	0 (0)	0 (0)	0.67 (1/1)	0 (0)	0 (0)	0.64 (1/1)	0 (0)	0 (0)
Peritoneal fluid (*N* = 1)	0.74 (1/1)	0 (0)	0 (0)	0.47 (1/1)	0 (0)	0 (0)	0.8 (1/1)	0 (0)	0 (0)	0.57 (1/1)	0 (0)	0 (0)
Hand carriage (*N* = 16)	0.82 ± 0.13 (10/15)	0 (0)	1 (0/1)	0.56 ± 0.2 (14/15)	0 (0)	0.44 (1/1)	0.76 ± 0.09 (14/15)	0 (0)	0.71 (1/1)	0.65 ± 0.11 (15/15)	0 (0)	0.63 (1/1)

N: number of tested isolates

n: number of isolates with positive activity for the corresponding hydrolytic enzyme

SD: standard deviation

### Phospholipase activity

Of the 172 isolates of *C. parapsilosis*, 111 (63.5%) were phospholipase positive. 92 (53.5.0%) had moderate activity. The mean Pz value for positive *C. parapsilosis* isolates was 0.85 ± 0.12. None of the *C. metapsilosis* or *C. orthopsilosis* isolates was able to produce phospholipase (Table 2). A significant difference in phospholipase activity was detected between *C. parapsilosis* and *C. metapsilosis* isolates (*P* = 0.028).

The phospholipase activity of *C. albicans* was statistically significantly higher than that of the *C. parapsilosis* complex species (*P* = 0.0001).

The strains isolated from blood culture, skin, and hand carriage of health workers showed similar Pz average values with no statistically significant differences observed among them (*P* > 0.668).

### Caseinase activity

A total of 157 (92.3%) isolates of *C. parapsilosis* were caseinase producers, most of which (87.2%) showed strong enzymatic activity. For *C. metapsilosis*, all isolates were proteinase positive, 2 (33.3%) of which were shown to be strong producers. For *C. orthopsilosis*, the four isolates (100%) were strong producers.

Higher caseinase activities were detected in *C. parapsilosis* (0.5 ± 0.18) and *C. orthopsilosis* (0.49 ± 0.07) than in *C. metapsilosis* isolates (0.72 ± 0.1) with a statistical significant difference (*P* = 0.012).

Most caseinase-producing *C. albicans* strains had strong (59.4%) or moderate (28.1%) enzymatic activity. Statistical significant difference was observed between the mean caseinase indices of *C. albicans* and *C. parapsilosis* complex species (*P* = 0.0001).

It was noted that a higher percentage of *C. parapsilosis* isolates recovered from blood (96.8%), skin (100%) and catheter (100%) were positive for caseinase activity (Table 3). No statistical significant difference was observed between the mean caseinase indices and the clinical site of isolation (*P* > 0.356).

### Gelatinase activity

The majority of tested fungal isolates showed gelatinase activity except for 6 among 172 *C. parapsilopsis* strains. 47.1% of *C. parapsilosis* and 50% of *C. metapsilosis* isolates exhibited low Pz values, which indicate high enzymatic production; whereas the majority of *C. albicans* isolates (78.1%) and *C. orthopsilosis* isolates (75%) displayed moderate Pz values.

No statistical significant difference was observed between the mean gelatinase Pz values of *C. albicans* and *C. parapsilosis* complex species (*P* = 0.698).

Gelatinase production was high in *C. parapsilosis* isolated from vaginal sample and respiratory tract (Pz =0.67). For *C. orthopsilosis* and *C. metapsilosis,* strains isolated from blood culture exhibited stronger activities with lowest average Pz values (Table 3).

### Haemolytic activity

All of the *C. albicans*, *C. parapsilopsis, C. metapsilosis* and *C. orthopsilosis* isolates had haemolytic activity on human blood SDA. The majority (79.7%) of *C. parapsilosis* isolates showed a strong activity. But, the majority of *C. metapsilosis* (83.3%) and *C. orthopsilosis* (75%) isolates showed moderate activities. Moreover, *C. metapsilosis* exhibited the low hemolysin production with statistical significant differences compared to *C. parapsilosis* (*P* = 0.0001) and *C. orthopsilosis* (*P* = 0.026).

The haemolytic activity of *C. albicans* was statistically significantly lower than that of the *C. parapsilosis* isolates (*P* = 0.0001).

But, no statistical significant difference was observed between the mean hemolysin production and the clinical site of isolation (*P* > 0.05) (Table 3).

### Biofilm formation

Table 4 presented the results of biofilm quantification using CV staining. Importantly, it was noticed that generally *C. parapsilosis* biofilms had more total biomass (average Abs_620_ = 0.475) compared with *C. orthopsilosis* (average Abs_620_ = 0.301) and *C. metapsilosis* (average Abs_620_ = 0.075).

**Table 4:** Biofilm production from *C. parapsilosis* complex species isolated from different clinical sites

	*C. parapsilosis*			*C. metapsilosis*			*C. orthopsilosis*		
	N	Mean Abs ± SD	Range	N	Mean Abs ± SD	Range	N	Mean Abs ± SD	Range
Blood (*N* = 64)	62	0.415 ± 0.625	0.009–3.093	1	0.055	0.055–0.055	1	0.304	0.304–0.304
Urine (*N* = 31)	29	0.770 ± 0.949	0.016–3.903	1	0.094	0.094–0.094	1	0.564	0.564–0.564
Auricular sample (*N* = 43)	39	0.347 ± 0.347	0.003–1.559	3	0.080 ± 0.056	0.031–0.142	1	0.191	0.191–0.191
Respiratory tract (*N* = 4)	4	0.804 ± 0.615	0.172–1.612	0	0	0	0	0	0
Catheter (*N* = 9)	9	0.611 ± 0.562	0.080–1.776	0	0	0	0	0	0
Skin (*N* = 8)	7	0.334 ± 0.372	0.044–1.070	1	0.061	0.061–0.061	0	0	0
Nails (*N* = 2)	2	0.953 ± 1.303	0.032–1.875	0	0	0	0	0	0
Oral cavity (*N* = 3)	3	1113 ± 0,718	0.283–1.547	0	0	0	0	0	0
Vagina (*N* = 1)	1	0.824	0.824–0.824	0	0	0	0	0	0
Peritoneal fluid (*N* = 1)	1	0.263	0.263–0.263	0	0	0	0	0	0
Hand carriage (*N* = 16)	15	0.182 ± 0.174	0.021–0.482	0	0	0	1	0.145	0.145–0.145
Total (*N* = 182)	172	0.475 ± 0.633	0.003–3.903	6	0.075 ± 0.038	0.031–0.142	4	0.301 ± 0.187	0.145–0.564

N: number of tested isolates

n: number of isolates with positive activity for the corresponding hydrolytic enzyme

SD: standard deviation

In contrast, *C. parapsilosis* strains were heterogeneous in terms of the level of biofilm formation with a range of 0.003–3.903 and a 1301-fold difference between the highest and lowest biofilm-producing strains. *C. metapsilosis* strains exhibited a more homogeneous behavior with all strains being low biofilm formers. But, no significant difference in biofilm-forming ability was detected between *C. parapsilosis*, *C. orthopsilosis* and *C. metapsilosis* isolates (*P* = 0.384).

The mean Abs_620_ value for the *C. albicans* strains was 0.421 (±0.504, SD) with a range of 0.075–2.033. No statistical significant difference was observed between biofilm formation of this species and *C. parapsilosis* complex species (*P* = 0.967).

There was no statistically significant association between biofilm-forming ability and the clinical origin of the isolates (*P* > 0.05).

Correlation analysis results with Pearson’s coefficient revealed a positive correlation between secretion of caseinase and hemolysin (*r* = 0.219, *P* ≤ 0.01). Moreover, biofilm production was correlated to secretion of gelatinase (*r* = 0.148, *P* ≤ 0.05). But, a negative correlation (*r* = −0.234, *P* ≤ 0.01) between, biofilm production and phospholipase production was detected.

### Antifungal susceptibility testing

The profiles of the in vitro susceptibility of *C. parapsilosis* complex species to to eight antifungal drugs are summarized in Table 5. According to the interpretative criteria for resistance used for the antifungal drugs described in the material and methods, we found that few isolates were resistant to azoles. Some *C. parapsilosis* isolates met the criterion for S-DD to fluconazole (10.91%), itraconazole (16.36%) and voriconazole (7.27%). Moreover, 5.45% and 1.82% of *C. parapsilosis* isolates were respectively resistant to fluconazole and voriconazole. All strains of *C. metapsilosis* and *C. orthopsilosis* were susceptible to azoles; and isolates of all three species exhibited 100% of susceptibility to caspofungin, amphotericin B and 5-flucytosine.

**Table 5:** In vitro susceptibility of *C. parapsilosis* complex species to eight antifungal drugs

	*C. parapsilosis* (*N* = 55)					*C. metapsilosis* (*N* = 6)					*C. orthopsilosis* (*N* = 4)				
	Range μg/ml	Mean μg/ml	S %	SDD %	R %	Range μg/ml	Mean μg/ml	S %	SDD %	R %	Range μg/ml	Mean μg/m	S %	SDD %	R %
Fluconazole	0.25–32	2.427	83.64	10.91	5.45	0.5–1	0.833	100	0	0	0.25–0.5	0.437	100	0	0
Itraconazole	0.008–0.5	0.096	83.64	16.36	0	0.016–0.125	0.078	100	0	0	0.016–0.064	0.052	100	0	0
Ketoconazole	0.008–16	0.321	ND	ND	ND	0.008–0.016	0.013	ND	ND	ND	0.008–0.016	0.010	ND	ND	ND
Posaconazole	0.008–1	0.106	ND	ND	ND	0.016–0.08	0.040	ND	ND	ND	0.032–0.08	0.052	ND	ND	ND
Voriconazole	0.008–8	0.190	90.91	7.27	1.82	0.008–0.032	0.018	100	0	0	0.008–0.016	0.010	100	0	0
Caspofungin	0.032–1.5	0.426	100	0	0	0.064–0.25	0.136	100	0	0	0.064–0.25	0.126	100	0	0
Amphotericin B	0.016–1	0.293	100	0	0	0.064–0.5	0.240	100	0	0	0.064–0.25	0.172	100	0	0
5-Flucytosine	0.03–2	0.110	100	0	0	0.03–0.03	0.030	100	0	0	0.03–0.064	0.055	100	0	0

*ND* Not determined due to lack of validated clinical breakpoints, *S* susceptible, *SDD* dose-dependent susceptible, *R* resistant

### Mechanisms of azole resistance

**Fig. 1:**
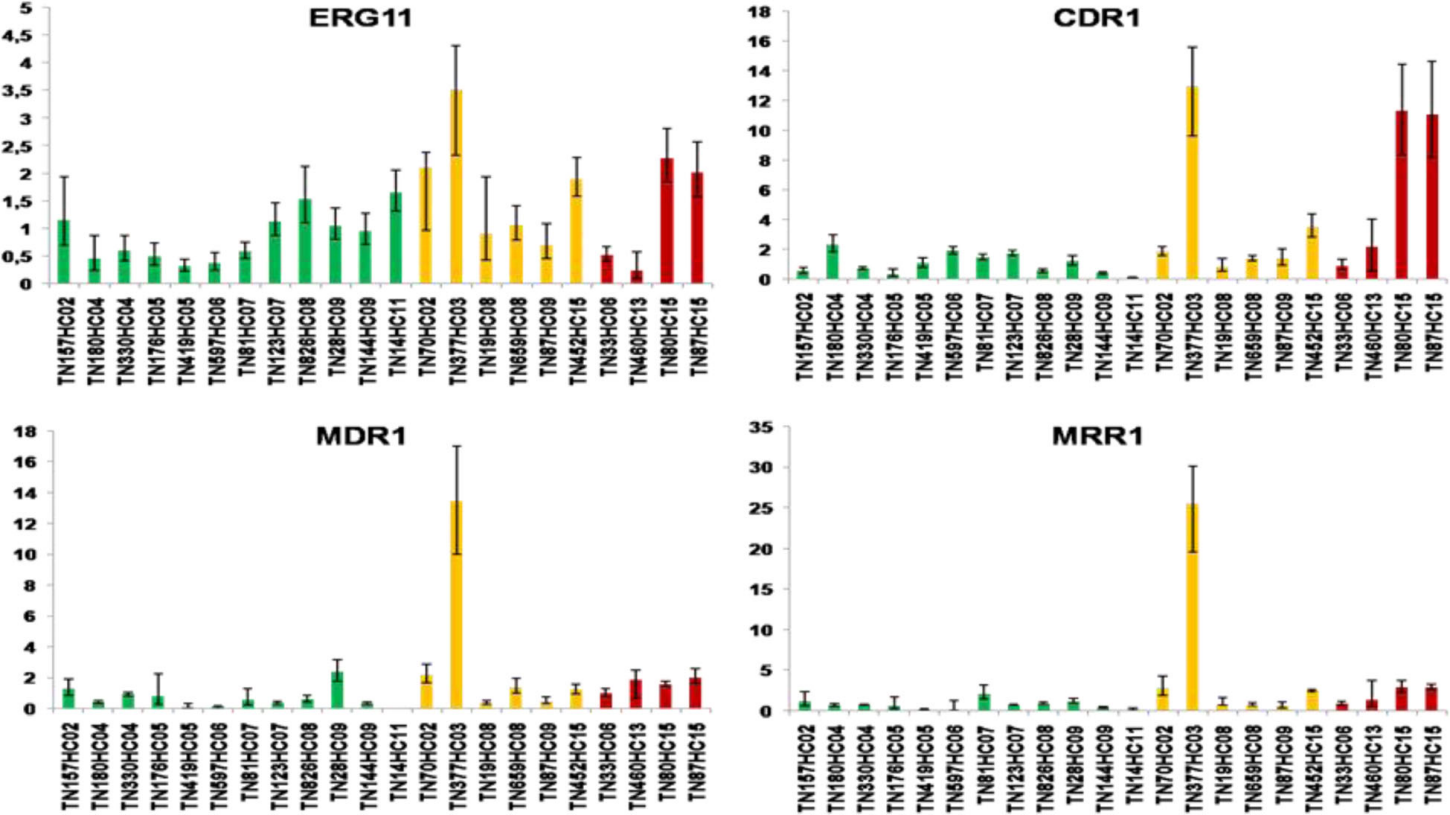
Study of the level of expression of ERG11, CDR1, MDR1 and MRR1 genes by relative quantification with RT-qPCR in *Candida parapsilosis*: 12 susceptible strains (green bars), 6 dose-dependent susceptible strains (yellow bars) and 4 resistant strains (red bars). Error bars represent the relative maximum (RQ max) and minimum (RQ min) quantifications with one standard deviation

We were also interested in elucidating the molecular mechanisms associated with the resistance of clinical strains of *C. parapsilosis* to azoles. For this we analyzed by RT-qPCR the quantitative expression and copy number of four genes (CDR1, MDR1 and MRR1) responsible for the efflux of the azoles and the ERG11 gene (target of the azoles) of four resistant strains, six dose- dependent susceptible strains and twelve susceptible strains isolated from blood culture, comparing to the ACT1 gene and the susceptible strain TN106HC05 (GenBank Accession number KX421285). The overexpression of one or more genes was observed in five (50%) of the 10 clinical isolates of dose-dependent susceptible and resistant *C. parapsilosis* isolates (Fig. 1). None of these genes was overexpressed in the strains susceptibles to the different azoles (Table 6). An overexpression of the CDR1 and MRR1 genes was noted in 2 out of 4 resistant strains and 2 out of 6 dose-dependent susceptible strains. In susceptible strains, the level of expression of CDR1, MRR1 and MDR1 varied respectively from 0.077 to 2.296, 0.052 to 2.108 and 0 to 2.352. The level of expression of CDR1 varied from 0.88 to 12.99 folds and the level of MRR1 varied from 0.558 to 25.498 in resistant and dose-dependent susceptible strains. The overexpression of the CDR1 and MRR1 genes was significantly associated with the resistant and dose-dependent susceptible phenotype (*P* = 0.015). The overproduction of the MDR1 gene was observed in a single dose-dependent susceptible strain with a level of expression equal to 13.401. Overexpression of the MRR1 gene was correlated with overexpression of the MDR1 gene only for a single strain (TN377HC03) among the four strains over-expressing the transcription factor MRR1. For the ERG11 gene, overproduction was observed also in the single isolate (TN377HC03) with a dose-dependent susceptible phenotype and it was expressed 3.518 folds. No upregulation of ERG11 was noted in susceptible strains. The overexpression of the CDR1, MDR1 and ERG11 genes was not associated with an increased copy number of gene in our strains of *C. parapsilosis*. However, the presence of MRR1 transcription factor in multiple copies at the genome has been associated with overexpression in two dose-dependent susceptible strains of *C. parapsilosis* (Table 6).

**Table 6:** Antifungal susceptibility to the azoles and relative quantification of gene expression and gene copy number of ERG11, CDR1, MDR1 and MRR1 genes in *Candida parapsilosis*

Strain	Posaconazole		Fluconazole		Itraconazole		Ketoconazole		Voriconazole		RNA relative quantification				DNA relative quantification			
	MIC	I	MIC	I	MIC	I	MIC	I	MIC	I	ERG11	CDR1	MDR1	MRR1	ERG11	CDR1	MDR1	MRR1
TN157HC02	0,016	ND	0,5	S	0,064	S	0,008	ND	0,008	S	1153	0,527	1259	1169	1367	2042	1087	3437
TN180HC04	0,032	ND	0,25	S	0,125	S	0,008	ND	0,008	S	0,445	2296	0,379	0,696	0,317	12,977	0,203	0,227
TN330HC04	0,032	ND	0,5	S	0,016	S	0,008	ND	0,008	S	0,595	0,693	0,901	0,733	6578	9570	6364	7141
TN176HC05	0,064	ND	1	S	0,064	S	0,016	ND	0,016	S	0,489	0,309	0,781	0,589	0,296	0,217	0,019	0,034
TN419HC05	0,016	ND	0,5	S	0,008	S	0,008	ND	0,008	S	0,314	1045	0,053	0,117	0,357	0,461	0,105	0,958
TN597HC06	0,032	ND	1	S	0,064	S	0,016	ND	0,016	S	0,367	1890	0,074	0,081	0,773	1613	0,247	3938
TN81HC07	0,032	ND	0,25	S	0,032	S	0,008	ND	0,008	S	0,579	1457	0,524	2108	1961	1597	3665	0,895
TN123HC07	0,032	ND	0,5	S	0,032	S	0,016	ND	0,032	S	1123	1729	0,327	0,731	0,973	0,766	1061	1971
TN826HC08	0,016	ND	1	S	0,064	S	0,008	ND	0,016	S	1525	0,538	0,584	0,909	1421	1220	1257	0,979
TN28HC09	0,08	ND	1	S	0,064	S	0,008	ND	0,016	S	1046	1196	2352	1166	0,929	1005	0,866	0,562
TN144HC09	0,125	ND	2	S	0,032	S	0,016	ND	0,016	S	0,948	0,378	0,312	0,364	0,502	1006	0,343	2949
TN14HC11	0,032	ND	0,25	S	0,064	S	0,008	ND	0,008	S	1644	0,077	0,000	0,052	0,261	1877	0,495	1913
TN70HC02	0,5	ND	4	SDD	0,5	SDD	0,064	ND	0,125	S	2115	1841	2192	2812	4434	7221	3060	13,487
TN377HC03	0,5	ND	4	SDD	0,25	SDD	0,125	ND	0,125	S	3518	12,990	13,401	25,498	0,506	2129	0,327	2977
TN19HC08	0,5	ND	4	SDD	0,25	SDD	0,064	ND	0,5	SDD	0,906	0,819	0,375	0,761	0,469	0,881	0,636	1659
TN659HC08	0,032	ND	4	SDD	0,5	SDD	0,125	ND	0,32	SDD	1055	1363	1381	0,725	1486	2482	0,878	0,907
TN87HC09	0,125	ND	4	SDD	0,032	S	0,016	ND	0,016	S	0,697	1365	0,472	0,558	0,503	0,617	0,303	2681
TN452HC15	0,032	ND	0,5	S	0,25	SDD	0,125	ND	0,125	S	1899	3504	1213	2489	0,411	1066	0,649	2711
TN33HC06	1	ND	16	R	0,5	SDD	0,25	ND	0,25	SDD	0,518	0,880	0,971	0,874	1792	7426	1342	10,307
TN460HC13	0,016	ND	0,5	S	0,016	S	16	ND	8	R	0,238	2184	1845	1313	0,568	1319	0,392	3028
TN80HC15	0,5	ND	32	R	0,25	SDD	0,032	ND	0,008	S	2277	11,320	1563	2886	3320	0,490	2572	1425
TN87HC15	0,5	ND	32	R	0,25	SDD	0,032	ND	0,008	S	2013	11,097	2016	2845	3472	0,444	2604	1262

*I* interpretation, *ND* Not determined due to lack of validated clinical breakpoints, *S* susceptible, *SDD* dose-dependent susceptible, *R* resistant, *MIC* in μg/ml

In addition, correlation analysis results with Pearson’s coefficient revealed that there was no statistically significant association between all virulence factors (biofilm-forming ability, gelatinase, caseinase, hemolysin, phospholipase) and the expression of the four genes (CDR1, MDR1 and MRR1) responsible for the efflux of the azoles and the ERG11 gene (*P* > 0.05).

## Discussion

Multiple virulence factors such as extracellular secreted hydrolytic and biofilm development have been developed by *Candida* species to assist in their ability to colonize host tissues, cause disease, and overcome host defenses. Since the report that *C. parapsilosis* was a cryptic complex of three species, many epidemiological studies have been reported worldwide. However, a less number of studies focused on biochemical/metabolic properties, antifungal susceptibilities, virulence factors’ expression and pathogenesis of the three specie [1, 13, 36, 64]. Moreover, some discrepancies were observed in findings among studies from different geographical regions. So, we conducted this study to compare the pathogenic potential of *Candida parapsilosis* complex species isolated from various clinical samples based on the examination of the following features: hydrolytic enzyme production, biofilm formation, and antifungal susceptibilities.

Extracellular hydrolytic enzymes seem to play an important role in candidal overgrowth, as these enzymes facilitate adherence and tissue penetration and hence invasion of the host [47]. Phospholipases facilitate the invasion of host mucosal epithelia by hydrolysing one/more ester linkages in glycerophospholipids, which are believed to be involved in disrupting the host cell membranes [45]. In our study, among the 172 isolates of *C. parapsilosis* 63.5% were phospholipase positive with moderate activity for the majority of strains. Similarly, Treviño-Rangel Rde J et al. reported that 63% of the *C. parapsilosis* sensu stricto isolates exhibited phospholipase activity, and 53% were strong producers [59]. Others studies founded low or undetectable phospholipase activity among *C. parapsilosis* isolates [1, 13, 36, 64]. Though, the majority of phospholipase- negative *C. parapsilosis* isolates was able to produce another lypolytic enzyme such esterase [1].

None of the *C. metapsilosis* or *C. orthopsilosis* isolates was able to produce phospholipase as it was reported by others studies [13]. However, Treviño-Rangel Rde J et al. described that almost all of the *C. orthopsilosis* (97%) and *C. metapsilosis* (80%) isolates showed phospholipase activity [59]. Moreover, Ge YP et al. showed that 90.5% of *C. parapsilosis* and 91.7% of *C. metapsilosis* isolates were phospholipase producers with high proportion of strong production [23].

This wide variation in phospholipase activity of *C. parapsilosis* complex species is of interest. It was attributed to the use of different media for enzymatic test, small sample size, and/or inherent biological variations among isolates… [1, 13, 23].

Additionally, contrasting findings were also reported in studies comparing systemic and superficial isolates. Some investigators found positive activity only in blood isolates [59] while others identified a higher level of activity among superficial isolates [23]. In our study, we don’t found an interesting statistical association between the clinical origin of the isolates, particularly those recovered from blood and phospholipase production. But, it was interesting to note that the strains isolated from blood culture, skin, and hand carriage of health workers showed similar activity. Therefore, phospholipase production can become a parameter to distinguish virulent invasive strains from non-invasive colonizers [12, 45].

Proteinases exhibit broad substrate specificity and are capable of degrading host epithelial and mucosal barrier proteins such as albumin, collagen, keratin, and mucin. They also aid *Candida* to resist cellular and humoral immunity by degrading antibodies, complement, and cytokines [7]. Most of the studies on exoenzymes produced by *C. parapsilosis* complex species are focused on secreted aspartyl proteinases (SAP) which are secreted in vitro when the organism is cultured in the presence of exogenous protein (usually bovine serum albumin) as the nitrogen source. However, variable protease activity (ranged from 17% to 100%) has been found by different authors among *C. parapsilosis* sensu stricto isolates [1, 13, 34, 56, 59]. In this study, we opted to use casein and gelatin as substrates for evaluation of proteinase activity. 92.3% of *C. parapsilosis* isolates were caseinase producers, most of which (87.2%) showed strong enzymatic activity. Limited phenotypic data pertaining to the caseinolytic activities of *C. parapsilosis* complex species are available. The only previous study of caseinolytic activity among *C. parapsilosis* complex displayed that only 5O% of *C. parapsilosis* sensu stricto produced it [64].

Moreover, previous studies reported variable proteinase activity among the two others species of the cryptic complex. Some authors reported that none of the *C. metapsilosis* or *C. orthopsilosis* isolates exhibited proteinase activity [13, 56]. Sabino R et al. showed that *C. orthopsilosis* were SAP producers, whereas *C. metapsilosis* were not [44]. Others recent reports found also a high proportion of isolates of both *C. metapsilosis* and *C. orthopsilosis* exhibiting protease activity [23, 34, 59]. Using casein as substrate, all ours isolates of *C. metapsilosis* and *C. orthopsilosis* were proteinase positive. Higher caseinase activities were detected in *C. parapsilosis* and *C. orthopsilosis* than in *C. metapsilosis* isolates with a statistical significant difference. Ziccardi M et al. reported that none of *C. orthopsilosis* isolates was caseinase producer [64].

Interestingly, we showed that 96.5% of *C. parapsilosis* strains and all isolates of *C. metapsilosis* and *C. orthopsilosis* produced gelatinase. But, none of *C. albicans*, *C. glabrata* and *C. krusei* isolates possessed the ability to hydrolyze gelatin in another study [41]. In the present data, statistical significant difference was observed between the mean indices of *C. albicans* and *C. parapsilosis* complex species for caseinase but not for gelatinase.

The ability to express proteinase enzymes not only varies among different species of *Candida* but also differs among the strains of same species isolated from different body sites [16, 42]. Corroborating these findings, De Bernardis F et al. identified positive proteolytic activity in all skin isolates of *C. parapsilosis* but none in blood ones [14]. Cassone A et al. also detected a higher proteolytic activity in vaginal *C. parapsilosis* isolates when compared with blood isolates [9]. Tosun I et al. described that urine-derived isolates have higher SAP activities [56]. However, we showed that no statistical significant association between anatomical origin and production of this virulence factor [55].

Haemolytic activity is another virulence factor exhibited by pathogenic microorganisms which permits growth in the host using as a source of iron the hemoglobin an iron-binding protein [30]. In this study, all the strains possessed the ability to show haemolysis on blood agar as it was reported by others investigations [1, 20]. Moreover, *C. metapsilosis* exhibited the low hemolysin production with statistical significant differences compared to *C. parapsilosis* and *C. orthopsilosis.* Treviño-Rangel Rde J et al. described that hemolysin activity was significantly more abundant in *C. orthopsilosis* (87%) than *C. parapsilosis* sensu stricto (67%) and *C. metapsilosis* (80%) [59]. Interestingly, we noted that the haemolytic activity of *C. albicans* was statistically significantly lower than that of the *C. parapsilosis* isolates, which contrasted with the finding of previous studies [12, 36].

Biofilm formation is considered a virulence factor due to the ability to confer resistance to antifungal therapy and protect the fungal cells from host immune responses [60]. It besides possibly being in *Candida* a key factor for the survival of these species, and may also be responsible for them being particularly well adapted to colonization of tissues and indwelling devices [50]. However, this issue is not completely clear, once the literature reports many controversial data about the biofilm-forming capacity of the three species of the *C. parapsilosis* complex [43]. In our study, these three species were able to form biofilm. This finding agrees with the results of previous studies [32, 43]. The species with the highest biofilm production was *C. parapsilosis*, followed by *C. orthopsilosis* and further by *C. metapsilosis* [32, 43]. Though, Lattif AA et al. reported that clinical isolates of *C. parapsilosis*, *C. metapsilosis*, and *C. orthopsilosis* were able to form biofilm with similar surface topography and architecture on abiotic surface (silicone disks) [28]. Some authors have reported that *C. orthopsilosis* and *C. metapsilosis* isolates are not able to produce biofilms in vitro [15, 51, 56].

In the present work, it was noted also that the biofilm forming ability of *C. parapsilosis* was highly strain dependent with important heterogeneity, which was less evident with both *C. orthopsilosis* and *C. metapsilosis*. Silva S et al. demonstrated that biofilm forming ability, structure and matrix composition are highly species dependent with additional strain variability occurring with *C. parapsilosis* [50]. Such findings undoubtedly reflect inherent physiological differences between strains and could have significance with respect to pathogenic potential [50]. So, investigations of biochemical and genetic mechanisms in biofilms are needed.

Interestingly, there was no statistically significant association between biofilm-forming ability and the clinical origin of ours isolates [13, 15, 50]. Others reports have demonstrated that blood biofilm production was linked to anatomical origin of isolates [55]. Nevertheless, it is noteworthy that several of these mentioned studies are not directly comparable because they differ in important aspects, such as the biofilm formation process, the methods to evaluate biofilm production (i.e., crystal violet staining, XTT reduction assays, or measured transmittance or absorbance without staining), and the criteria for considering an isolate as a biofilm producer [13].

Interestingly, we found a statistically significant inverse correlation between phospholipase activity and the ability to form biofilm. This suggests that phospholipids are directly implicated in biofilm composition and stability, for *C. parapsilosis* complex species. According to Lattif AA et al., *candida* biofilms contained significantly higher levels of phospholipid and sphingolipids than planktonic cells and lipid rafts are critical to the ability of *Candida* to form biofilm [27].

Overall, our findings demonstrate that *C. metapsilosis* was the least virulent species of the *psilosis* group except for gelatinase activity, which was supported by the literature. In fact, according to the studies conducted in vitro or in vivo, *C. metapsilosis* has been reported as a less virulent member of the *C. parapsilosis* complex in an epithelial and epidermal tissue models [21], in an in vitro infection model using microglial cells [35], in a murine model of vaginal candidiasis [5] and also in an in vivo model system using *Galleria mellonella* larvae [34].

There is a growing concern related to antifungal susceptibility profiles of *C. parapsilosis* complex species. In this investigation, all *C. parapsilosis* isolates were susceptible to amphotericin B, and 5-flucytosine. Nevertheless, Some *C. parapsilosis* isolates met the criterion for S-DD to fluconazole (10.91%), itraconazole (16.36%) and voriconazole (7.27%). 5.45% and 1.82% of *C. parapsilosis* isolates were respectively resistant to fluconazole and voriconazole with some strains displayed a multiazole resistant phenotype [43, 49, 58]. These results are discordant with several studies demonstrating the greater efficacy of these new triazoles (i.e., voriconazole) against *C. parapsilosis* complex isolates [3].

According to the recently revised CBPs, none of our isolates were resistant to echinocandins [6, 55, 57]. Our data are inadequate to others surveys who described a reduced susceptibility to echinocandins probably due to a naturally occurring Proline to Alanine amino acid change (P660A) in the glucan synthase enzyme Fks1p [3, 22, 58]. Moreover, all *C. orthopsilosis* and *C. metapsilosis* were sensitive to the others tested drugs. In contrast, some reports have shown that these two species exhibited low susceptibilities to amphotericin B [17, 43], to fluconazole [11, 13, 24, 53] and to itraconazole [3, 43]. However, because of the small number of isolates belonging to the newly identified species, our study may not provide an entirely accurate picture of the antifungal susceptibility patterns of *C. parapsilosis* complex species. So, testing more isolates is required for determination of virtual rates of resistance among the strains of the two former species.

Despite the potentially rising incidence of *C. parapsilosis* and the threat that fluconazole resistance could pose in a clonally expanding population, previous studies have provided limited information concerning molecular mechanisms of azole resistance in *C. parapsilosis* [25, 48, 52, 63]. Quantification of drug resistance gene expression in *Candida* isolates with reduced azole susceptibility is a valuable tool for understanding the molecular mechanism(s) of azole resistance and monitoring for the emergence of resistance [29]. To our knowledge, this is the first assessment at molecular level of azole resistance mechanisms in *C. parapsilosis* isolates from Tunisia. We assessed the quantitative expression of the ABC transporter CDR1*,* the MFS transporters MDR1*,* the zinc cluster transcription factor MRR1*,* and also ERG11.

In our study, we confirmed the involvement of drug transporters CDR1 and MDR1 in the phenomenon of azole resistance in strains. Our findings showed an increase in CDR1 expression in 50% of the resistant strains and 33.3% of the dose-dependent susceptible strains, suggesting that this transporter contributes to the azole resistance. According to Berkow EL et al. (2015), sixteen strains of *C. parapsilosis* resistant isolates (45.7%) showed an increased CDR1 expression with a minimum of 2-fold [4]. In contrast, all resistant isolates of *C. parapsilosis* expressed increased levels of CDR1 (3.3–9.2 fold) in the presence of fluconazole during the study of Souza AC et al. (2015); but with a varied expression of the two drug transporters genes. More isolates showed CDR1 overexpression than MDR1overexpression [52]. In addition, overexpression of CDR1 and CDR2 has been shown to lead to cross-resistance of the same isolate to multiple azole antifungals, whereas overexpression of MDR1 has been associated with fluconazole resistance only [19, 61]. It has also been reported that CDR1 is more closely associated with azole resistance than CDR2 in *C. glabrata* [46] and in *C. albicans* [10].

Berkow EL et al. (2015) have suggested that the overexpression of the putative drug transporters CDR1 was due to activating mutations in the genes encoding their transcriptional regulators. In fact, among 16 CDR1-overexpressing isolates, mutations G650E and L978 W leading to amino acid substitutions were detected in TAC1 in respectively 2 isolates and 1 isolate. None of these SNPs corresponded to a documented activating mutation in CaTAC1 of *C. albicans* [4]. Silva AP et al. (2011) suggested an overexpression of CDR1 in fluconazole induced resistant *C. parapsilosis* strains, since they observed upregulation of the transcription factor NDT80 which, in *C. albicans,* modulates azole tolerance by controlling the expression of the CDR1 gene [48].

For *C. parapsilosis*, Berkow EL et al. (2015) observed a marked overexpression of the major facilitator efflux pump MDR1 in only three (out of 35) resistant isolates with a minimum 25-fold increase [4]. The same was found to be true for the isolates of *C. parapsilosis* examined in the present study which showed an overexpression of MDR1 for only one strain. In addition, in the study realized by Souza AC et al. (2015), 2 of 9 isolates showed increased mRNA expression of MDR1 in the presence of fluconazole [52]. However, MDR1 expression was upregulated by 19.43 and 40.22 folds in the resistant strains obtained after exposure to fluconazole and to voriconazole [48]. As for *C. albicans,* overexpression of MDR in *C. parapsilosis* is also correlated with increased expression of aldo-keto reductases and other genes associated with the oxidative stress response, which may protect cells from damage caused by toxic molecules produced in the presence of azoles and also contribute to antifungal drug resistance. The expression of these genes is also regulated by MRR1 transcription factor [48].

The present study showed that the expression of MRR1 was also upregulated in *C. parapsilosis*. Silva AP et al. (2011) also correlated the overexpression of MDR1 and mutations within MRR1 with fluconazole resistance in resistant isolates of *C. parapsilosis* [48]. More recently, a surveillance study of a collection of clinical isolates of *C. parapsilosis* has again implicated MDR1 and MRR1 in resistance to fluconazole in this species [4]. As reported for *C. albicans*, upregulation of MRR1 may be caused by single gain-of-function mutations [18, 33]. Two mutations (G1747A and A2619C) were identified in the MRR1 coding sequence of azole-resistant *C. parapsilosis* isolates that resulted in an amino acid exchange (G583R and K873 N) [8, 48]. According to Zhang L et al. (2015), overexpression of MDR1 genes were detected in the two resistant isolates, and this was associated with a homozygous mutation in MRR1 genes (T2957C /T2957C), with the amino acid exchange L986P [63]. According to Grossman NT et al. (2015), polymorphisms in MRR1 are common, and only some are associated with overexpression of MDR1. They suggest that there is a hot spot for gain-of-function mutations in MRR1, in the region coding from amino acids 852 to 875.They identified one clinical isolate with a polymorphism in this region, corresponding to L779F (G2337 T), which has 73-fold upregulation of MDR1 [25].

The upregulation of the ERG11 gene was noted only in one dose-dependent susceptible isolate from this collection. However, in the study of Berkow EL et al. (2015), ERG11 was found to be overexpressed in many of the azole-resistant clinical *C. parapsilosis* isolates, as has been observed in *C. albicans*. Eight isolates (22.8%) exhibited a minimum increase in ERG11 expression from 2-fold to 11-fold in *C. parapsilosis* [4]. Silva AP et al. (2011) founded that expression of ERG11 was reduced in the resistant strains of *C. parapsilosis* [48]. This observation might be related to the fact that the ERG11 overexpression was assessed in the absence of exposure to azoles, likewise in our study [52]. Overexpression of ERG genes is correlated with increased expression of the transcription factors UPC2 and NDT80. In fact, a resistant strain obtained after exposure to posaconazole showed upregulation of these two transcription factors (UPC2 and NDT80) and increased expression of 13 genes involved in ergosterol biosynthesis [48].

Other resistance mechanisms apart from those already described, such as mutations in the target enzymes, might be implicated [8, 48]. Grossman NT et al. (2015) also examined the sequences of ERG11 for the presence of amino acid substitutions. They identified the Y132F substitution as well and in fact observed it in 56.7% of their fluconazole- resistant isolates. They concluded that this mutation is perhaps largely responsible for most of the fluconazole resistance observed within this species [25].

Contrary to what was expected, the upregulation of the CDR1, MDR1 and ERG11 genes was also not associated with an increased copy number of gene in our strains of *C. parapsilosis*. Thus, the presence of genes that encode membrane transporters in multiple copies at the genome of *C. parapsilosis* was not responsible for an increase in transcription levels in our isolates. Thus, in these strains the upregulation of these genes is controlled by another molecular mechanism.

## Conclusion

In conclusion, this investigation provides more information about the frequency of the production of the major enzymes considered to be virulence factors of *C. parapsilosis* complex species and reinforce the heterogeneity of this fungal complex. But, it still remains unclear what virulence factors may play a role in the final outcome. Moreover, the fact that phenotypic properties were found to significantly differ in strains isolated from various geographical regions suggests that other mechanisms such as epigenetic modifications may be used by this yeast to adapt to environmental changes [55]. Further in vivo studies are necessary and the molecular mechanisms of pathogenicity should be more explored, to better understand the pathogenesis of the infections caused by the *C. parapsilosis* species complex. Finally, we have demonstrated that a combination of molecular mechanisms, including the overexpression of ERG11, and genes encoding efflux pumps are involved in azole resistance in *C. parapsilosis*. However, it is likely that the presence of point mutations in the ERG11 gene or additional mutations in transcription factors, or other mechanisms still unknown, probably exist in ours strains of *C. parapsilosis*.

## References

[1] Abi-Chacra EA, Souza LO, Cruz LP, Braga-Silva LA, Goncalves DS, Sodre CL, Ribeiro MD, Seabra SH, Figueiredo-Carvalho MH, Barbedo LS, Zancope-Oliveira RM, Ziccardi M, Santos AL (2013). Phenotypical properties associated with virulence from clinical isolates belonging to the *Candida parapsilosis* Complex. FEMS Yeast Res.

[2] Aneeja K. Experiments in microbiology, plant pathology, tissue culture and mushroom cultivation, ed II edn New Age international Publishers, Delhi 1996.

[3] Ataides FS, Costa CR, Souza LK, Fernandes O, Jesuino RS, Silva Mdo R (2015). Molecular identification and antifungal susceptibility profiles of *Candida parapsilosis* Complex species isolated from culture collection of clinical samples. Rev Soc Bras Med Trop.

[4] Berkow EL, Manigaba K, Parker JE, Barker KS, Kelly SL, Rogers PD (2015). Multidrug transporters and alterations in sterol biosynthesis contribute to azole antifungal resistance in *Candida parapsilosis*. Antimicrob Agents Chemother.

[5] Bertini A, De Bernardis F, Hensgens LA, Sandini S, Senesi S, Tavanti A (2013). Comparison of *Candida parapsilosis, Candida orthopsilosis,* and *Candida metapsilosis* adhesive properties and pathogenicity. Int J Med Microbiol.

[6] Borghi E, Sciota R, Iatta R, Biassoni C, Montagna MT, Morace G (2011). Characterization of *Candida parapsilosis* Complex strains isolated from invasive fungal infections. Eur J Clin Microbiol Infect Dis.

[7] Borst A, Fluit AC (2003). High levels of hydrolytic enzymes secreted by *Candida albicans* isolates involved in respiratory infections. J Med Microbiol.

[8] Branco J, Silva AP, Silva RM, Silva-Dias A, Pina-Vaz C, Butler G, Rodrigues AG, Miranda IM (2015). Fluconazole and Voriconazole resistance in *Candida parapsilosis* is conferred by gain-of-function mutations in MRR1 transcription factor gene. Antimicrob Agents Chemother.

[9] Cassone A, De Bernardis F, Pontieri E, Carruba G, Girmenia C, Martino P, Fernandez-Rodriguez M, Quindos G, Ponton J (1995). Biotype diversity of *Candida parapsilosis* and its relationship to the clinical source and experimental pathogenicity. J Infect Dis.

[10] Chen LM, Xu YH, Zhou CL, Zhao J, Li CY, Wang R (2010). Overexpression of CDR1 and CDR2 genes plays an important role in fluconazole resistance in *Candida albicans* with G487T and T916C mutations. J Int Med Res.

[11] Chen YC, Lin YH, Chen KW, Lii J, Teng HJ, Li SY (2010). Molecular epidemiology and antifungal susceptibility of *Candida parapsilosis* Sensu stricto, *Candida orthopsilosis,* and *Candida metapsilosis* in Taiwan. Diagn Microbiol Infect Dis.

[12] Chin VK, Foong KJ, Maha A, Rusliza B, Norhafizah M, Ng KP, Chong P (2013). *P. candida albicans* isolates from a Malaysian hospital exhibit more potent phospholipase and haemolysin activities than non-albicans *Candida* isolates. Trop Biomed.

[13] da Silva BV, Silva LB, de Oliveira DB, da Silva PR, Ferreira-Paim K, Andrade-Silva LE, Silva-Vergara ML, Andrade AA (2015). Species distribution, virulence factors, and antifungal susceptibility among *Candida parapsilosis* complex isolates recovered from clinical specimens. Mycopathologia.

[14] De Bernardis F, Mondello F, San Millan R, Ponton J, Cassone A (1999). Biotyping and virulence properties of skin isolates of *Candida parapsilosis*. J Clin Microbiol.

[15] de Toro M, Torres MJ, Maite R, Aznar J (2011). Characterization of *Candida parapsilosis* Complex isolates. Clin Microbiol Infect.

[16] Deorukhkar SC, Saini S (2015). Virulence factors attributed to pathogenicity of non albicans *Candida* species isolated from human immunodeficiency virus infected patients with oropharyngeal candidiasis. Annals of pathology and Lab Med.

[17] Diekema DJ, Messer SA, Boyken LB, Hollis RJ, Kroeger J, Tendolkar S, Pfaller MA (2009). In vitro activity of seven systemically active antifungal agents against a large global collection of rare *Candida* species as determined by CLSI broth microdilution methods. J Clin Microbiol.

[18] Dunkel N, Blass J, Rogers PD, Morschhauser J (2008). Mutations in the multi-drug resistance regulator MRR1, followed by loss of heterozygosity, are the main cause of MDR1 overexpression in fluconazole-resistant *Candida albicans* strains. Mol Microbiol.

[19] Frade JP, Warnock DW, Arthington-Skaggs BA (2004). Rapid quantification of drug resistance gene expression in *Candida albicans* by reverse transcriptase LightCycler PCR and fluorescent probe hybridization. J Clin Microbiol.

[20] Franca EJ, Furlaneto-Maia L, Quesada RM, Favero D, Oliveira MT, Furlaneto MC (2011). Haemolytic and proteinase activities in clinical isolates of *Candida parapsilosis* and *Candida tropicalis* with reference to the isolation anatomic site. Mycoses.

[21] Gacser A, Schafer W, Nosanchuk JS, Salomon S, Nosanchuk JD (2007). Virulence of *Candida parapsilosis, Candida orthopsilosis,* and *Candida metapsilosis* in reconstituted human tissue models. Fungal Genet Biol.

[22] Garcia-Effron G, Katiyar SK, Park S, Edlind TD, Perlin DSA (2008). Naturally occurring proline-to-alanine amino acid change in Fks1p in *Candida parapsilosis, Candida orthopsilosis,* and *Candida metapsilosis* accounts for reduced echinocandin susceptibility. Antimicrob Agents Chemother.

[23] Ge YP, Lu GX, Shen YN, Liu WD (2011). In vitro evaluation of phospholipase, proteinase, and esterase activities of and *Candida metapsilosis*. Mycopathologia.

[24] Gomez-Lopez A, Alastruey-Izquierdo A, Rodriguez D, Almirante B, Pahissa A, Rodriguez-Tudela JL, Cuenca-Estrella M (2008). Prevalence and susceptibility profile of *Candida metapsilosis* and *Candida orthopsilosis*: results from population-based surveillance of candidemia in Spain. Antimicrob Agents Chemother.

[25] Grossman NT, Pham CD, Cleveland AA, Lockhart SR (2015). Molecular mechanisms of fluconazole resistance in *Candida parapsilosis* isolates from a U.S. surveillance system. Antimicrob Agents Chemother.

[26] Jiang C, Dong D, Yu B, Cai G, Wang X, Ji Y, Peng Y (2013). Mechanisms of azole resistance in 52 clinical isolates of *Candida tropicalis* in China. J Antimicrob Chemother.

[27] Lattif AA, Mukherjee PK, Chandra J, Roth MR, Welti R, Rouabhia M, Ghannoum MA (2011). Lipidomics of *Candida albicans* biofilms reveals phase-dependent production of phospholipid molecular classes and role for lipid rafts in biofilm formation. Microbiology.

[28] Lattif AA, Mukherjee PK, Chandra J, Swindell K, Lockhart SR, Diekema DJ, Pfaller MA, Ghannoum MA (2010). Characterization of biofilms formed by *Candida parapsilosis, C. metapsilosis,* and *C. orthopsilosis*. Int J Med Microbiol.

[29] Li QQ, Skinner J, Bennett JE (2012). Evaluation of reference genes for real-time quantitative PCR studies in *Candida glabrata* following azole treatment. BMC Mol Biol.

[30] Linares CE, de Loreto ES, Silveira CP, Pozzatti P, Scheid LA, Santurio JM, Alves SH (2007). Enzymatic and hemolytic activities of *Candida dubliniensis* strains. Rev Inst Med Trop Sao Paulo.

[31] Luo G, Samaranayake LP, Yau JY (2001). *Candida* species exhibit differential in vitro hemolytic activities. J Clin Microbiol.

[32] Melo AS, Bizerra FC, Freymuller E, Arthington-Skaggs BA, Colombo AL (2011). Biofilm production and evaluation of antifungal susceptibility amongst clinical *Candida spp.* isolates, including strains of the Candida Parapsilosis Complex. Med Mycol.

[33] Morschhauser J, Barker KS, Liu TT, Bla BWJ, Homayouni R, Rogers PD (2007). The transcription factor Mrr1p controls expression of the MDR1 efflux pump and mediates multidrug resistance in *Candida albicans*. PLoS Pathog.

[34] Nemeth T, Toth A, Szenzenstein J, Horvath P, Nosanchuk JD, Grozer Z, Toth R, Papp C, Hamari Z, Vagvolgyi C, Gacser A (2013). Characterization of virulence properties in the *C. parapsilosis* Sensu Lato species. PLoS One.

[35] Orsi CF, Colombari B, Blasi E (2010). *Candida metapsilosis* as the least virulent member of the '*C. parapsilosis*' complex. Med Mycol.

[36] Pakshir K, Zomorodian K, Karamitalab M, Jafari M, Taraz H, Ebrahimi H (2013). Phospholipase, esterase and hemolytic activities of *Candida spp.* isolated from onychomycosis and oral lichen planus lesions. J Mycol Med.

[37] Park BJ, Arthington-Skaggs BA, Hajjeh RA, Iqbal N, Ciblak MA, Lee-Yang W, Hairston MD, Phelan M, Plikaytis BD, Sofair AN, Harrison LH, Fridkin SK, Warnock DW (2006). Evaluation of amphotericin B interpretive breakpoints for *Candida* bloodstream isolates by correlation with therapeutic outcome. Antimicrob Agents Chemother.

[38] Pfaller MA, Chaturvedi V, Diekema DJ, Ghannoum MA, Holliday NM, Killian SB, Knapp CC, Messer SA, Miskou A, Ramani R (2012). Comparison of the Sensititre YeastOne colorimetric antifungal panel with CLSI microdilution for antifungal susceptibility testing of the echinocandins against *Candida spp.*, using new clinical breakpoints and epidemiological cutoff values. Diagn Microbiol Infect Dis.

[39] Pfaller MA, Chaturvedi V, Diekema DJ, Ghannoum MA, Holliday NM, Killian SB, Knapp CC, Messer SA, Miskov A, Ramani R (2008). Clinical evaluation of the Sensititre YeastOne colorimetric antifungal panel for antifungal susceptibility testing of the echinocandins anidulafungin, caspofungin, and micafungin. J Clin Microbiol.

[40] Price MF, Wilkinson ID, Gentry LO (1982). Plate method for detection of phospholipase activity in *Candida albicans*. Sabouraudia.

[41] Ramesh N, Priyadharsini M, Sumathi CS, Balasubramanian V, Hemapriya J, Kannan R (2011). Virulence factors and anti fungal sensitivity pattern of *Candida Sp.* isolated from HIV and TB patients. Indian J Microbiol.

[42] Ramos Lde S, Barbedo LS, Braga-Silva LA (2015). Dos Santos a.L., pinto M.R. And Sgarbi D.B. Protease and phospholipase activities of *Candida spp.* isolated from cutaneous candidiasis. Rev Iberoam Micol.

[43] Ruiz LS, Khouri S, Hahn RC, da Silva EG, de Oliveira VK, Gandra RF, Paula CR (2013). Candidemia by species of the *Candida parapsilosis* Complex in children's hospital: prevalence, biofilm production and antifungal susceptibility. Mycopathologia.

[44] Sabino R, Sampaio P, Carneiro C, Rosado L, Pais C (2011). Isolates from hospital environments are the most virulent of the *Candida parapsilosis* Complex. BMC Microbiol.

[45] Sachin CD, Ruchi K, Santosh S (2012). *In vitro* evaluation of proteinase, phospholipase and haemolysin activities of *Candida* species isolated from clinical specimens. Int J Med Biomed Res.

[46] Sanguinetti M, Posteraro B, Fiori B, Ranno S, Torelli R, Fadda G (2005). Mechanisms of azole resistance in clinical isolates of *Candida glabrata* collected during a hospital survey of antifungal resistance. Antimicrob Agents Chemother.

[47] Schaller M, Borelli C, Korting HC, Hube B (2005). Hydrolytic enzymes as virulence factors of *Candida albicans*. Mycoses.

[48] Silva AP, Miranda IM, Guida A, Synnott J, Rocha R, Silva R, Amorim A, Pina-Vaz C, Butler G, Rodrigues AG (2011). Transcriptional profiling of azole-resistant *Candida parapsilosis* strains. Antimicrob Agents Chemother.

[49] Silva AP, Miranda IM, Lisboa C, Pina-Vaz C, Rodrigues AG (2009). Prevalence, distribution, and antifungal susceptibility profiles of *Candida parapsilosis, C. orthopsilosis,* and *C. metapsilosis* in a tertiary care hospital. J Clin Microbiol.

[50] Silva S, Henriques M, Martins A, Oliveira R, Williams D, Azeredo J (2009). Biofilms of non-*Candida albicans Candida* species: quantification, structure and matrix composition. Med Mycol.

[51] Song JW, Shin JH, Shint DH, Jung SI, Cho D, Kee SJ, Shin MG, Suh SP, Ryang DW (2005). Differences in biofilm production by three genotypes of *Candida parapsilosis* from clinical sources. Med Mycol.

[52] Souza AC, Fuchs BB, Pinhati HM, Siqueira RA, Hagen F, Meis JF, Mylonakis E, Colombo A (2015). *L. candida parapsilosis* resistance to fluconazole: molecular mechanisms and in vivo impact in infected galleria mellonella larvae. Antimicrob Agents Chemother.

[53] Szabo Z, Szilagyi J, Tavanti A, Kardos G, Rozgonyi F, Bayegan S, Majoros L (2009). In vitro efficacy of 5 antifungal agents against , *Candida orthopsilosis,* and *Candida metapsilosis* as determined by time-kill methodology. Diagn Microbiol Infect Dis.

[54] Tavanti A, Davidson AD, Gow NA, Maiden MC, Odds F (2005). *C. candida orthopsilosis* and *Candida metapsilosis spp. nov.* to replace Candida Parapsilosis groups II and III. J Clin Microbiol.

[55] Tavanti A, Hensgens LA, Mogavero S, Majoros L, Senesi S, Campa M (2010). Genotypic and phenotypic properties of *Candida parapsilosis* Sensu strictu strains isolated from different geographic regions and body sites. BMC Microbiol.

[56] Tosun I, Akyuz Z, Guler NC, Gulmez D, Bayramoglu G, Kaklikkaya N, Arikan-Akdagli S, Aydin F (2013). Distribution, virulence attributes and antifungal susceptibility patterns of *Candida parapsilosis* Complex strains isolated from clinical samples. Med Mycol.

[57] Trabasso P, Matsuzawa T, Fagnani R, Muraosa Y, Tominaga K, Resende MR, Kamei K, Mikami Y, Schreiber AZ, Moretti ML (2015). Isolation and drug susceptibility of *Candida parapsilosis* Sensu Lato and other species of *C. parapsilosis* Complex from patients with blood stream infections and proposal of a novel LAMP identification method for the species. Mycopathologia.

[58] Trevino-Rangel Rde J, Garza-Gonzalez E, Gonzalez JG, Bocanegra-Garcia V, Llaca JM, Gonzalez GM (2012). Molecular characterization and antifungal susceptibility of the *Candida parapsilosis* species complex of clinical isolates from Monterrey, Mexico. Med Mycol.

[59] Trevino-Rangel Rde J, Gonzalez JG, Gonzalez GM (2013). Aspartyl proteinase, phospholipase, esterase and hemolysin activities of clinical isolates of the *Candida parapsilosis* species complex. Med Mycol.

[60] Tumbarello M, Posteraro B, Trecarichi EM, Fiori B, Rossi M, Porta R, de Gaetano Donati K, La Sorda M, Spanu T, Fadda G, Cauda R, Sanguinetti M (2007). Biofilm production by *Candida* species and inadequate antifungal therapy as predictors of mortality for patients with candidemia. J Clin Microbiol.

[61] Vandeputte P, Larcher G, Berges T, Renier G, Chabasse D, Bouchara JP (2005). Mechanisms of azole resistance in a clinical isolate of *Candida tropicalis*. Antimicrob Agents Chemother.

[62] White TJ B.T., Lee S, Taylor J. Amplification and direct sequencing of fungal ribosomal RNA genes for phylogenetics.. PCR protocols: a guide to methods and applications.:315–322., 1990.

[63] Zhang L, Xiao M, Watts MR, Wang H, Fan X, Kong F, Development XYC (2015). Of fluconazole resistance in a series of *Candida parapsilosis* isolates from a persistent candidemia patient with prolonged antifungal therapy. BMC Infect Dis.

[64] Ziccardi M, Souza LO, Gandra RM, Galdino AC, Baptista AR, Nunes AP, Ribeiro MA, Branquinha MH, Santos A (2015). *L. candida parapsilosis* (sensu lato) isolated from hospitals located in the southeast of Brazil: species distribution, antifungal susceptibility and virulence attributes. Int J Med Microbiol.

